# Gait Alterations in Adults after Ankle Fracture: A Systematic Review

**DOI:** 10.3390/diagnostics12010199

**Published:** 2022-01-14

**Authors:** Marta Mirando, Corrado Conti, Federica Zeni, Fabio Pedicini, Antonio Nardone, Chiara Pavese

**Affiliations:** 1 Centro Studi Attività Motorie of Pavia Institute, Istituti Clinici Scientifici Maugeri IRCCS, 27100 Pavia, Italy; marta.mirando@icsmaugeri.it; 2 Department of Clinical-Surgical, Diagnostic and Pediatric Sciences, University of Pavia, 27100 Pavia, Italy; corrado.conti01@universitadipavia.it (C.C.); federica.zeni01@universitadipavia.it (F.Z.); chiara.pavese@unipv.it (C.P.); 3 Department of Translational Medicine, Università del Piemonte Orientale, 13100 Vercelli, Italy; fabio.pedicini@unipv.it; 4 Neurorehabilitation and Spinal Units of Pavia Institute, Istituti Clinici Scientifici Maugeri IRCCS, 27100 Pavia, Italy

**Keywords:** ankle fracture, gait analysis, rehabilitation

## Abstract

(1) Background: Ankle fracture results in pain, swelling, stiffness and strength reduction, leading to an altered biomechanical behavior of the joint during the gait cycle. Nevertheless, a common pattern of kinematic alterations has still not been defined. To this end, we analyzed the literature on instrumental gait assessment after ankle fracture, and its correlation with evaluator-based and patient-reported outcome measures. (2) Methods: We conducted a systematic search, according to the Preferred Reporting Items for Systematic Reviews and Meta-analyses guidelines, of articles published from January 2000 to June 2021 in PubMed, Embase and PEDro on instrumental gait assessment after ankle fracture. (3) Results: Several changes in gait occur after ankle fracture, including a reduction in step length, swing time, single support time, stride length, cadence, speed and an earlier foot-off time in the affected side. Additionally, trunk movement symmetry (especially vertical) is significantly reduced after ankle fracture. The instrumental assessments correlate with different clinical outcome measures. (4) Conclusions: Instrumental gait assessment can provide an objective characterization of the gait alterations after ankle fracture. Such assessment is important not only in clinical practice to assess patients’ performance but also in clinical research as a reference point to evaluate existing or new rehabilitative interventions.

## 1. Introduction

The anatomical definition of ankle refers to the joint formed by the tibia, fibula, and talus, which is physiologically stabilized by the action of neighbor muscles and ligaments. However, most of the surgical and functional classifications of ankle fractures include lesions of tibial and/or fibular malleolus and the neighboring ligamentous structures, but not talus fractures, which are usually classified as foot injuries. The three most used ankle fracture classifications are that of Danis–Weber, based on the level of the fibula fracture, that of Lauge-Hansen, focused on the mechanism of injury, and the Arbeitsgemeinschaft für Osteosynthesefragen Foundation/Orthopedic Trauma Association (AO/OTA) classification, based on the location of the fracture lines and the degree of comminution [1].

Ankle fractures represent one of the most common causes of access to a Trauma Center, with an annual incidence of 107 to 184 per 100,000, accounting for about 9% of all fractures [2,3]. Malleoli are the structures most frequently involved: about 60–70% of ankle fractures are unimalleolar (mainly affecting the lateral malleolus), followed by bimalleolar (15–20%) and trimalleolar fractures (7–12%) [4]. The etiology of ankle fracture commonly involves a traumatic event, but the diverse dynamics can result in different clinical presentations. There are several risk factors for ankle fracture: sports practice (especially basketball, football, soccer and skiing), elderly age [5], high body mass index (BMI), smoking [6], one or more falls in the previous year, alcohol consumption, living alone, average sleep time <7 h/day, osteoarthritis, family history of hip fracture and reduced bone mineral density [7,8]. Males are more predominant in the younger age groups, females more predominant in the older age groups [9].

Treatment of ankle fracture is based on radiological findings, individual patient characteristics, and the clinical presentation, and hence may involve different conservative or surgical approaches. After surgery or plaster cast removal, rehabilitation begins: the combination of impaired active and passive range of motion, reduced muscle strength, altered proprioception and pain as a consequence of both fracture and treatment requires a personalized rehabilitation program involving ankle mobility, strengthening exercises, weight bearing and balancing exercises [10].

In addition to the body structure impairment, ankle fracture results in a modification of the biomechanical behavior of the joint, thus compromising the movements of the entire limb during the gait cycle [11]. The alteration of the gait cycle is highly relevant in rehabilitation, since it may expose the patient to modification of the load distribution on different joints, pain and increased risk of fall [12].

Impairment after ankle fracture is evaluated in clinical practice through outcome assessment questionnaires and scales, which report patients’ and evaluators’ assessment of recovery. However, the questionnaires commonly used in clinical practice are unable to capture the fine alterations of biomechanical behavior of the joint. For this, instrumental methods of gait assessment can provide objective and reproducible information about the alteration of gait.

**Figure 1:**
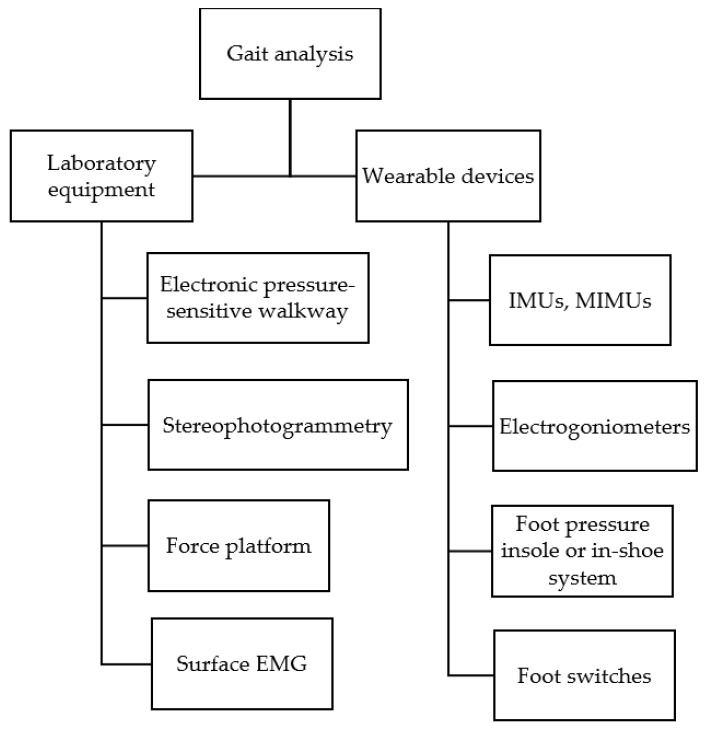
Diagram presenting the main techniques for gait analysis. One or more wearable devices can also be used in a laboratory setting.

Figure 1 outlines the main methods used to objectively study gait. Instrumental methods for gait analysis include systems designed for use in a laboratory setting as well as wearable devices, i.e., usable both indoors and outdoors. In the laboratory setting, stereophotogrammetry, force platforms and electronic pressure-sensitive walkways are commonly used for gait analysis. Wearable devices include inertial measurement units (IMUs), magnetic and inertial measurement units (MIMUs), foot switches, foot pressure insole or in-shoe systems, surface electromyography (EMG) and electrogoniometers. According to the Italian Society of Clinical Movement Analysis (SIAMOC) position paper, gait analysis in a clinical context should include surface EMG recording in addition to stereophotogrammetry and force platforms [13].

Stereophotogrammetry is performed by detecting either active (light-emitting) or passive (retroreflective) markers, fixed at appropriate body landmarks, through infrared cameras. This enables a 3-D reconstruction of the body movements and joint angles (kinematics) [14]. All spatial and temporal variables of gait can thus be obtained. Two or more force platforms embedded in a walkway make it possible to simultaneously record ground reaction forces during the stance phase of gait and measure the displacement of the center of pressure (CoP), i.e., the point of application of the resultant ground reaction forces [15], providing an estimation of joint moments (kinetics). In turn, surface EMG allows us to measure spatial and temporal activation of several muscles involved in gait as well as their intensity of activation [16]. In addition, EMG recording can help to interpret the reduced joint motion [17], e.g., to determine if it is due to insufficient muscle activation or to cocontraction.

Gait analysis can also include measurement of the plantar pressure distribution [18]. This is obtained using an electronic pressure-sensitive walkway or a foot pressure insole/in-shoe system—the terms used in different studies vary: e.g., baropodometry, pedobarography, pedography, plantar pressure measurement. These instruments detect the pressure interface: the electronic pressure-sensitive walkway, between the foot and the walkway; the foot pressure insole/in-shoe system, between foot and footwear. It is thus possible to measure the pressure distribution in the different segments of the footsole during the stance phase of gait as well as the displacement of the CoP, as in the case of force platforms.

Gait analysis is commonly used to assist diagnosis and treatment of gait abnormalities, inform surgical procedures, and evaluate treatment effects [19]. Nowadays, results obtained from gait analysis performed according to specific guidelines [13] may support clinical decision making in the case of gait disability [20]. Unfortunately, gait analysis requires large spaces and specifically trained personnel, is time consuming, and cannot provide information about walking performance in daily life.

In order to overcome these limitations and to allow an ecological assessment of gait, in recent decades wearable devices have been introduced in the quantitative assessment of gait. Whole IMUs consist of accelerometers and gyroscopes; when equipped with magnetometers, these devices are called MIMUs. IMUs detect linear acceleration and angular velocity of the body segment to which they are attached, whilst MIMUs detect in addition the magnetic north, allowing one to estimate the direction or, so-called, heading. IMUs and MIMUs can be used alone or in combination with some of the above instrumental methods for gait analysis. In particular, using adaptive algorithms to process data in a short time, IMUs fixed at different body segments can provide a real-time estimate of spatiotemporal variables of gait and joint ROM as well as of trunk displacement. IMUs have opened the way to clinical applications in different diseases as well as measurement of activities of daily living [21]. The foot pressure insole/in-shoe system can be used not only indoors, as outlined above, but also outdoors, and during different tasks of daily living. It can be useful to assess the interface pressure between the footsole and the footwear, whether normal or an orthopedic shoe. Foot switches are low-cost sensors, usually fixed at the heel, and first and fifth metatarsal heads of each foot. They detect foot contact with the ground and are not usually employed in gait analysis per se but rather to validate the algorithms used with different types of sensors [22]. Electrogoniometers, either rigid or flexible, allow 2-D measurement of joint angles during gait [23,24]. Their use in gait analysis has declined in recent years in favor of IMUs.

The above instrumental methods of gait assessment may be useful in recovery after ankle fracture to correlate the severity of musculoskeletal impairment with biomechanical function and to monitor patients’ progress over the course of rehabilitation and postrehabilitation, for clinical and research purposes [25,26]. However, to our knowledge, a common pattern of the kinematic alterations of gait after ankle fracture has still not been defined. Hence, the aim of this systematic review was to explore the evidence in the literature on the instrumental evaluation of gait after ankle fracture and investigate how well the gait variables correlate with evaluator-based scoring systems and patient-reported outcome measures, in an attempt to characterize the pattern of gait alterations after ankle fracture.

## 2. Materials and Methods

A systematic search was performed in July 2021 by two authors independently (C.C. and F.Z.) in PubMed, Embase and PEDro electronic databases for peer-reviewed scientific literature. The aim was to explore the current use of instrumental evaluation of gait after ankle fractures, searching the literature from January 2000 to June 2021. Ankle fractures were defined according to the Lauge-Hansen classification, one of the most widely used ankle fracture classification systems [27,28].

In PubMed and Embase we screened titles/abstracts in the English language using the following terms:

“fracture* AND (ankle OR malleol* OR plafond OR ‘distal tibia*’ OR ‘distal peron*’ OR ‘distal fibula*’ OR ‘tibiofibular syndesmosis’ OR ‘deltoid ligament’) AND (gait OR rehabilit*)”

For the research in PEDro, we combined the same research words. We then performed a data crosscheck to delete duplicate articles obtained from each database.

### 2.1. Eligibility Criteria

We included in the review all articles concerning instrumental evaluation of gait after ankle fracture that met the following criteria: (a) published in English language; (b) published since year 2000; (c) involving adults > 18 years of age, thus excluding articles enrolling children; (d) directly investigating human subjects, i.e., excluding simulation; (e) research articles, i.e., excluding congress acts; (f) all types of study design except systematic and narrative reviews. 

### 2.2. Selection Process and Intervention

The selection process was performed by the same two authors independently by reading the title and abstract of all studies and applying the criteria mentioned above. Then, the two authors compared the included and excluded studies to assess differences. In cases of discordance, if title and abstract did not give enough information according to the inclusion/exclusion criteria, the full text was retrieved, evaluated, and discussed among all authors to arrive at a consensus. For each article included in the present study, the full text was retrieved, read, and analyzed by all authors. The selection process conformed to the Preferred Reporting Items for Systematic reviews and Meta-Analyses (PRISMA) statements [29].

### 2.3. Data Collection and Quality Assessment

Data extraction and quality assessment were independently carried out by two authors (C.C. and M.M.). For this systematic review, we collected the following information concerning the instrumental evaluations used: device (type and location) and gait variables assessed; in addition, we collected all clinical assessment tools applied in combination with the instrumental evaluation, including both evaluator-based scoring systems and patient-reported outcome measures.

Since most of the studies analyzed were single-arm trials, we applied the Methodological Index for Nonrandomized Studies (MINORS) scale to assess methodologic quality [30]. For noncomparative studies, the MINORS scale evaluates 8 aspects that qualify the methodological level of the study: a clearly stated aim, inclusion of consecutive patients, prospective data collection, appropriate endpoints, unbiased assessment of the study endpoint, appropriate follow-up time, number of patients lost to follow-up less than 5%, and prospective calculation of the study size. The additional criteria in the case of comparative studies include adequate control group, contemporary groups, baseline equivalence of groups, and adequate statistical analyses. Each MINORS item is scored as 0 (not reported), 1 (reported but inadequate), or 2 (reported and adequate). The optimal total score is 16 for noncomparative studies and 24 for comparative studies.

We assessed the methodological quality of the randomized controlled trials (RCTs) using the PEDro scale, which applies only to experimental studies but does not evaluate their clinical usefulness [31]. The PEDro scale evaluates 11 criteria, 10 of which are scored. For each criterion met by the study, 1 point is assigned (yes = 1, no = 0). Points are totaled to give a score out of 10. The methodological quality of the studies is usually categorized as follows: a score from 6 to 10 indicates high quality, of 4–5, moderate quality; and ≤3, low quality. Considering the small number of RCTs included in this review, assessing publication bias was deemed inappropriate.

As this review represents a qualitative summary of gait analysis after ankle fracture, the total score of the quality assessment was not used as an exclusion criterion for article selection.

## 3. Results

### 3.1. Study Selection and Quality Assessment

**Figure 2:**
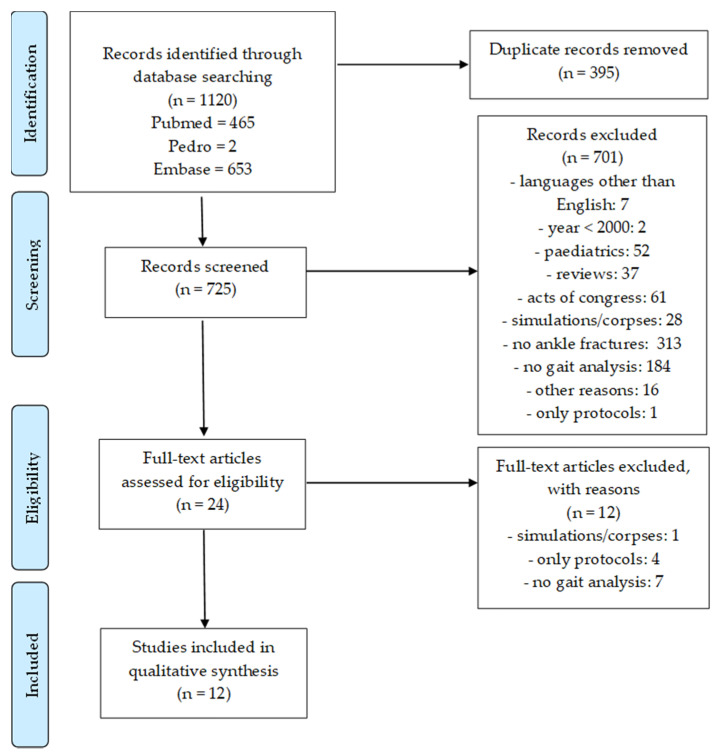
PRISMA flow diagram representing the search strategy and the selection of articles.

The literature search identified a total of 1120 studies: 465 from PubMed, 653 from Embase, and 2 from PEDro. After removing duplicate articles, we screened 725 studies for suitability, of which 12 studies were finally selected for the review. Figure 2 reports the PRISMA flow chart of the selection process.

Table 1 reports the quality assessment of the nonrandomized studies included according to the MINORS scale, while Table 2 reports the quality assessment of the two RCTs included according to the PEDro scale. Only 2 of the 10 nonrandomized studies reached a score of 16 points, indicating high methodological quality. Both RCT studies had a score in the top range (6–10 points), indicating high quality.

### 3.2. Synthesis of the Studies Included

Table 3 summarizes the information extracted from the 12 selected articles, with reference to patient characteristics, study design, use of orthosis, device used for gait analysis and variables assessed, additional clinical or instrumental evaluations and main results of gait analysis. Table 4 summarizes the domains and the clinical outcome measures analyzed in the selected articles in addition to instrumental evaluations.

A few studies applied an instrumental evaluation to define the characteristics of gait after ankle fracture and correlated the instrumental evaluations with clinical outcome measures.

In a prospective study, Hsu and colleagues [33] compared a sample of 10 patients with different types of ankle fracture treated with cast immobilization with or without open reduction and internal fixation (ORIF) with 10 age- and sex-matched healthy controls. Using an IMU constituted by a triaxial accelerometer fixed on the lower back (L3–L4), they found that at about 4 months after injury patients still showed altered gait, i.e., lower walking speed, step length and cadence. In addition, trunk acceleration root mean square (RMS) in anterior–posterior and vertical directions, as well as trunk movement symmetry in vertical direction, were significantly reduced. Conversely, no significant difference between the two subject groups was observed regarding regularity of trunk movement and acceleration RMS ratio (RMSR) in the mediolateral direction. The authors showed a correlation of gait and trunk movements with the Lower Extremity Functional Scale (LEFS) score. In particular, step length and walking speed showed a significant correlation with LEFS score. Acceleration RMSR in the mediolateral direction and stride regularity showed a correlation with risk of falls 24 months after fracture. 

Suciu and colleagues [37] compared 30 patients with bimalleolar fractures who underwent surgery and rehabilitation with 21 healthy controls. They analyzed the changes in temporal and spatial gait variables and functional outcomes. The study group was assessed twice: at weight-bearing concession (T1) and 12 weeks after an exercise-based rehabilitation program (T2). Each evaluation consisted of a functional questionnaire (Olerud–Molander Ankle Score, OMAS) and analysis of temporal and spatial gait variables. The following variables were analyzed using a treadmill with an integrated electronic pressure-sensitive walkway: step time, stride time, step length, stride length, stance time, swing time, load response time, pre swing time, single support time, cadence, and speed. At T1, all temporal and spatial gait variables were reduced in the patient group, in both the affected and nonaffected ankle. At T2, an improvement was observed: indeed, there were no significant differences in patients compared to controls in terms of step time in the affected and nonaffected ankle, swing time and stance time on the affected ankle, or stride time and cadence. However, when compared to the nonaffected ankle, step time, step length, stance, swing, and single support time were significantly shorter in the affected leg at both T1 and T2. The OMAS improved significantly from T1 to T2 in all subscales except for squatting.

Jansen and colleagues [34] studied the long-term (19 to 100 months) clinical outcome and changes in gait of 35 patients with unilateral tibial pilon fracture surgically treated. Clinical outcome was assessed by the American Orthopedic Foot and Ankle Society (AOFAS) score, the visual analogue scale (VAS) foot and ankle scale, and the Phillips score. To evaluate the changes in gait, the authors used an electronic pressure-sensitive walkway, analyzing load, pressure and force–time integral. Goniometry highlighted a reduction in ROM in the affected side compared with the healthy side involving both extension/flexion and pronation/supination movements. The VAS score correlated with the fracture pattern according to the AO classification. Both AOFAS and Phillips scores showed an inverse correlation with the AO classification: lower AO values were associated with better clinical outcomes. The three questionnaires showed that patients had a satisfactory functional status. Baropodometry showed lateralization of weight bearing on the injured foot. Indeed, the injury side showed less loading and a lower force–time integral in the heel region and under the first metatarsal region, but a higher loading and higher force–time integral under the fourth and fifth metatarsal. The authors showed that clinical outcome according to the different questionnaires and onset of post-traumatic arthrosis correlated with fracture severity in the AO classification. Moreover, lateralization of weight bearing correlated with clinical outcome.

Segal and colleagues [36] conducted a case–control observational study to analyze the effects of malleolar fractures on gait and functional performance compared to healthy controls. Forty-one patients were surgically treated with ORIF according to Arbeitsgemeinschaft für Osteosynthesefragen/Association of the Study of Internal Fixation (AO/ASIF) methods [2], followed by a period of 6 weeks of weight-bearing prohibition. Each participant underwent only one baropodometric evaluation, performed when weight bearing was allowed. All three fracture-severity groups were significantly below the normal range in all gait variables including gait speed, involved and uninvolved step length, and involved/uninvolved single-limb support. Walking speed was significantly higher in patients with unimalleolar ankle fracture compared to bimalleolar, but not trimalleolar, fracture. Patients with unimalleolar fracture showed a significantly longer step length in the uninvolved leg compared with the bimalleolar and trimalleolar groups. Additionally, single-limb support time was significantly longer in the unimalleolar ankle fracture group compared to both the bimalleolar and trimalleolar groups. A significant asymmetry in step length and single-limb support in all fracture groups, but not in healthy controls, was also found. Significantly higher AOFAS scores were found in the unimalleolar fracture group compared to the bimalleolar and trimalleolar fracture groups, but the difference between the bimalleolar and trimalleolar fracture groups was not significant. Finally, the study highlighted a significant direct proportionality between the fracture severity and distance walked in the 6-min walk test: patients with unimalleolar fracture walked the longest distance, those with trimalleolar fracture the shortest. Analyzing the Short Form (SF-36) Health Survey scores revealed no significant difference among the ankle fracture groups.

A similar perspective, single-center, case–control, level II evidence study was conducted by Van Hoeve and colleagues [40], who performed gait analysis in patients with surgically treated ankle fractures. Thirty-three patients were recruited at, on average, 18 months (range 7–57) postsurgery. Gait analysis was performed on a 10 m walkway with a force platform positioned in the middle, using stereophotogrammetry following the Oxford Foot Model [3,4,5]. The investigators found a significant difference between fracture and control groups when subjects were asked to walk at their preferred normal speed. Patients showed reduced ROM at flexion/extension of the ankle joint during the loading and push-off phases, but not at abduction/adduction nor at inversion/eversion movements. Patients with trimalleolar fractures reported significantly lower ROM at the flexion/extension movements compared to unimalleolar patients; the trimalleolar fracture group also had the lowest Foot and Ankle Disability Index (FADI) and AOFAS scores. The AOFAS ankle–hindfoot score showed a significant correlation with the ankle flexion/extension ROM during both the push-off and the loading phase. The authors highlighted a reduced flexion and extension of the ankle joint in patients with more severe fracture, although no significant differences in ROM were found between unimalleolar and bimalleolar fracture patients. Patients with trimalleolar ankle fractures compared to healthy subjects showed a significant reduction in ankle ROM.

The case–control study of Elbaz and colleagues [32] compared lower limb gait kinematics of 24 patients after recovery from an ankle fracture injury with the same number of healthy controls. Patients were recruited within 6 weeks following weight-bearing approval. Knee and hip ROM were estimated from IMUs placed bilaterally on the lateral side of the calf and thigh. Compared to healthy controls, patients showed lower knee ROM during the swing and stance phases. Moreover, the study highlighted a reduction in lower thigh and calf ROM during the whole gait cycle in both the involved and uninvolved limb and a longer stride duration compared to healthy controls. Within the patient group, no significant differences in symmetry were found in any other measurement. 

The case–control study of Wang and colleagues [11] used stereophotogrammetry to analyze the foot motion changes at 12 months after ORIF, according to the AO principle [6], in 18 patients with ankle fractures. Patients, asked to walk at self-selected speed, showed less plantarflexion in the fractured joint compared to the nonfractured one. Additionally, the big toe showed less dorsiflexion. In turn, the forefoot of the affected leg showed less plantarflexion and adduction in the swing phase than the nonfractured side in comparison to control subjects. Regarding spatiotemporal gait variables, stride length and single support time were decreased, and foot-off time occurred earlier on the fractured side than on the nonfractured one. Finally, a significant correlation between OMAS and kinematic variables in the sagittal plane was also found.

Prescription and/or orthosis footwear plays an important role in the treatment of ankle and foot pathologies. In spite of this, we found only four studies that evaluated the effect of prescription footwear and orthoses after ankle fracture using instrumental methods of gait assessment. 

In their case–control study, Terrier and colleagues [38] used a triaxial accelerometer fixed on the lower back (L3–L4) to investigate the improvement of gait (stride symmetry and regularity) with orthopedic shoes in 16 patients with ankle and/or foot fractures: eight patients were equipped with low shoes (standard orthopedic shoes) and eight with ankle boots (stabilizing shoes). Gait was evaluated comparing the results in each patient with vs. without orthopedic shoes, as well as between the two patient groups with either type of shoe, and, finally, with healthy controls. In patients with orthopedic shoes, stride symmetry and stride regularity significantly increased, and pain VAS score decreased. Without orthopedic shoes, stride symmetry of patients was lower than that of 16 healthy subjects, whilst stride regularity did not differ from that of healthy subjects.

In a subsequent observational study, Terrier and colleagues [39] recruited 25 patients with persistent post-traumatic impairments and disability 8 months after ankle and/or foot fracture. They analyzed the changes in trunk accelerations in anteroposterior, vertical and mediolateral directions (detected by a triaxial accelerometer fixed at the lower back) induced by orthopedic shoes. Patients received orthopedic shoes with custom-made orthoses (insoles). Three patients were equipped with ankle boots, one with open shoes and 21 with low shoes; all shoes were equipped with rocker soles. The authors calculated the local dynamic stability in order to assess the ability (with vs. without orthopedic shoes) to attenuate the effects of small local perturbations on gait and keep it smooth. The results showed that orthopedic shoes significantly improved local dynamic stability in the three axes. Conversely, cadence was not different with orthopedic shoes. In addition, footwear adaptation led to pain relief and improved foot and ankle proprioception. Finally, the average AOFAS score increased at the end of hospitalization.

Keene and colleagues [42] performed a randomized crossover trial to compare the effect of three different ankle supports (walker boot, stirrup-brace and elasticated tubular bandage) on gait characteristics and pain in 18 patients. The following gait variables were recorded through an electronic pressure-sensitive walkway: step-length and single-limb support time asymmetry (between injured and uninjured limbs), step width and gait speed. The evaluation was performed 6 weeks after ankle internal fixation surgery, one hour after cast removal. The study highlighted a statistically significant but clinically not meaningful faster speed with the walker boot than with the bandage. Compared to the bandage, there was no evidence of an effect of stirrup-brace use on walking speed at any test speed. With increasing walking speed, asymmetry between limbs decreased in terms of step length and single support time, and step width narrowed. No difference in step-length asymmetry between limbs was observed when walking with the bandage compared to the walker boot or stirrup brace. Furthermore, asymmetry in single support time between limbs was reduced by 3% in the stirrup brace and by 5% in the walker boot. Step width was 1.2 cm wider when walking with the walker boot. There was no difference in step width between the bandage and the stirrup brace. Pain was immediately reduced with the use of the walker boot, and to a lesser extent, with the stirrup brace compared to the tubular bandage. 

Quacinella and colleagues [35] sought to determinate whether an ankle–foot orthosis (AFO) would improve gait variables (speed, cadence, stride length, and single-leg stance duration) and pain in seven patients with pilon fractures. Gait variables were analyzed with stereophotogrammetry and force platforms. Spatiotemporal gait data were measured before and after AFO application. With AFO, median gait speed significantly improved (from 1.1 m/s to 1.3 m/s). No other variables showed significant modification. Nor was the AFO associated with any improvement in pain.

We found only one study that investigated with instrumental evaluations the effect of manual therapy after ankle fracture. Albin and colleagues [41] conducted a double-blind randomized clinical trial to evaluate the short-term effect of manual therapy on ankle ROM, triceps surae muscle stiffness, gait, and balance in patients undergoing ORIF of an ankle or hindfoot fracture. Seventy-two participants had received previous physical therapy consisting of balance exercise and gait training. Patients were advised to continue their home exercise program, but not to add any new exercise; they were then randomized into two groups: the study group received impairment-based manual therapy, the control group received sham manual therapy consisting of light soft-tissue mobilization and proximal tibiofibular joint mobilizations. Participants completed the outcome measures at baseline, after their second visit, and 7–10 days later. Gait was assessed with an electronic pressure-sensitive walkway, recording gait speed, percent of time spent in single-limb support and stance time. The two groups showed no difference in terms of motion, gait, or balance when comparing baseline assessment with final follow-up. There was no change in gastrocnemius muscle stiffness in the manual therapy group, whereas in the control group’s muscle stiffness increased in a relaxed (prone) but not in a contracted (performing a heel raise) position.

## 4. Discussion

### 4.1. Main Findings

Our review analyzed the literature concerning the use of instrumental methods in evaluating kinetic and kinematic variables of gait after ankle fracture. The study highlights several changes in gait after ankle fracture, including a reduction in step length, swing time, single support time, stride length, cadence, and speed, and an earlier foot-off time in the affected side [33,34,35,37,40]. These gait alterations are unspecific since they can be due to several causes including pain, weakness, stiffness, swelling or alteration of proprioception [43].

Normal gait is characterized by a high degree of trunk movement symmetry and regularity, which means low trunk movement variability between each step or stride [44]. After ankle fracture, trunk movement symmetry, particularly in a vertical direction, is significantly lower in patients with ankle fracture. Moreover, trunk acceleration in the mediolateral axis correlated with falls occurring during a 24-month follow-up after ankle fracture [33]. The causes of changes in trunk movements are multiple: reduced plantar flexor moment at the affected ankle joint could interfere with heel contact; weakness of lower trunk muscles, e.g., iliopsoas and gluteal muscles, could lead to poor control of vertical acceleration of the center of gravity during the loading and midstance phases; decreased range of motion, reduced peak muscle torque, and atrophy of ankle muscles following immobilization after fracture might be related to displacement of center of mass in the sagittal plane, consequently interfering with between-steps trunk movement symmetry in the vertical axis [45].

A few studies reported ROM limitations in the different segments of the injured lower limb in the sagittal plane [11,32]. Limitations include a reduction in knee ROM during the swing phase, of maximum knee flexion angle during stance, as well as of thigh and calf ROM through a single gait cycle with respect to healthy subjects. In particular, the reduced maximum knee flexion angle of the affected limb during stance might indicate less loading time on the involved limb [32]. Furthermore, muscle atrophy could also affect gait in patients following ankle fracture, given the proximity of measurements to the no-load period [46].

Ankle ROM in the frontal plane (abduction/adduction) and transversal plane (inversion/eversion) did not show significant differences during the loading phase or the push-off phase. On the other hand, ROM between the hindfoot and tibia in the sagittal plane (flexion/extension) during both loading and push-off phases was lower in patients with ankle facture. This finding seems to correlate with the increasing severity of the fracture [40].

The injured side showed a reduction in sagittal and transversal ROM in the forefoot, and reduced sagittal ROM in the hindfoot and hallux segments. The injured side was found to have less plantarflexed hindfoot, forefoot, and less dorsiflexed hallux during pre-swing than the noninjured side [11]. These differences in gait reflect a clear movement limitation and suggest that patients after ankle fracture may adopt avoidance behavior during gait. Indeed, a clearly disturbed walking pattern of the injured side was confirmed also by reduction in load, pressure, and force–time integral in the heel region and under the first metatarsal region, which suggests a lateralization of load bearing on the injured limb [34].

### 4.2. Findings in the Context of the Literature

The ankle joint has a complex structure: its solidity, even during the full weight-bearing phase of gait cycle, is explained by the stabilizing cooperation of numerous muscles and ligaments. Similarly to other parts of the body, rarely are ankle fractures isolated: they are frequently associated with ligament and tibiofibular syndesmosis lesions, articular cartilage damage, and dislocation of bone fragments [43]. Due to the bone and ligament lesions and also to the surgical or conservative treatment, the normal passive and active range of motion of the ankle may be reduced, thus compromising the biomechanical movements of the entire limb during the gait cycle. Pain sensation can have a further detrimental impact on body stability as well as on walking disability. 

Regarding ankle fractures, lab-based gait analysis allows us to accurately measure ankle and foot kinematics: hindfoot relative to tibia, forefoot relative to hindfoot, forefoot relative to tibia, and hallux relative to forefoot [11,35,40]. However, gait analysis performed in a laboratory setting requires, in addition to expensive instrumentation, trained personnel to collect and analyze the data in what is typically a time-consuming process [47]. This restricts the routine use of gait analysis to clinical and research facilities. On the contrary, wearable devices such as IMUs are portable and affordable, and provide a common alternative to the expensive and strictly lab-based methods of quantifying gait [21]. Electronic pressure-sensitive walkways and trunk accelerometry with IMUs have been demonstrated to be a simplified method to study gait variables after ankle fractures [32,38] that save the high costs and logistic problems linked to the gait laboratory. However, IMUs cannot directly measure spatial variables of gait and electronic pressure-sensitive walkways cannot be used to measure joint motion, which could add useful information and better reflect the patient’s condition. 

Lower extremity fractures have many associated complications and require long rehabilitation times [48]. Proper limb loading ensures a correct healing process with better fracture outcomes, including a decrease in time to union, a reduced number of complications, and improved functional outcomes. However, during the initial stages of healing, excessive mechanical loading or an unstable mechanical environment have a negative effect on bone healing [49]. At the same time, prolonged non-limb loading is associated with delayed healing and worse outcome [50]. However, micromovements, even at early stages, have been shown to significantly decrease the fracture’s healing time; also, a progressive increasing of loading during rehabilitation reduces the length of the healing period [51].

Early weight bearing for ankle fractures is supported by many authors [52,53] but no consensus has been reached on the rate of load to apply to optimize fracture healing. Furthermore, none of the studies we analyzed implemented use of pressure-sensitive insoles to evaluate limb loading during gait. Previous studies have shown that patients voluntarily restrict limb load in the first phase after lower extremity trauma, progressively increasing the amount of weight bearing over time [54]. Completely unsupervised weight bearing can lead to an increased risk of secondary fracture dislocation due to extensive overloading of the fracture (depending on the type) [55]. Moreover, teaching patients weight-bearing skills is known to be quite challenging. Using a foot pressure insole, with continuous monitoring of the compliance to weight-bearing protocols, seems to be an interesting tool to personalize weight-bearing prescription during the postoperative aftercare phase for clinical and research purposes [56,57].

Prescription footwear plays an important role in the treatment of ankle and foot pathologies. Following complex injuries, orthotic insoles and orthopedic shoes are designed to achieve joint stability by controlling the motion of foot and ankle, reducing the shock and shear, correcting malalignment, improving the foot rockers, and relieving pain by off-loading specific areas [58,59]. One of the main goals of footwear is to improve gait quality. The results of this review show that prescription of orthopedic shoes increased gait symmetry and reduced pain after ankle facture as well as improving stability of gait [39]. However, gait recovery after treatment is highly variable among patients. Consequently, the assessment of footwear outcome requires rapid, objective, and reproducible methods, which can evaluate gait more thoroughly than by simple observation. Trunk acceleration measurements, using accelerometry, might be of some help for clinicians to better evaluate the suitability of footwear prescription assessing gait symmetry and regularity. Overall, trunk accelerometry seems to be a simple tool able to give global gait quality indicators with good repeatability [38].

Functional outcome studies have been performed among patients with ankle fractures, analyzing the relationship between radiographic findings and physical examination as well as patient-reported outcome measures (PROMs). The application of PROMs is necessary to capture patients’ perception of impairment, disability and handicap. To this end, the availability of translated and validated instruments plays a crucial role for the assessment of patient-perceived problems after lower limb diseases [60,61]. The studies analyzed reported that patients experienced little or mild pain and few restrictions in functional activities one year after ankle fracture surgery [62]. Several studies examined the association between fracture severity and functional outcome, with mixed results. Some authors concluded that fracture classification can be used as a predictor of functional outcome after surgery [63,64]. As evidenced in the studies by Segal et al. [36] and Van Hoeve et al. [40], a correlation exists between functional scores and the number of malleoli involved by the fracture. Other authors found a significant correlation between fracture severity (in terms of the number of fractured malleoli and the AO classification) and several PROMS, in particular the SF-36 physical functioning and AOFAS ankle–hindfoot scores [40]. On the other hand, other authors did not find a correlation between the type of fracture and functional outcomes after surgery [65].

The instrumental evaluation with gait analysis highlighted that ankle kinematics are decreased in patients with severe ankle fractures; in particular, decreased ankle flexion and extension were found in patients with increased severity of fracture. The OMAS was found to be moderately correlated with kinematic variables in the sagittal plane, e.g., hindfoot/tibia peak dorsiflexion and ROM in the swing phase [11]. Additionally, performance of physical activities, evaluated by the LEFS score, was significantly correlated with walking speed and step length [33]. Based on these findings, it seems that the use of kinematic analysis in addition to clinical scales may help to refine the predictive capacity of PROMs.

### 4.3. Strengths and Limitations

To our knowledge, this is the first review to summarize the evidence in the literature on alterations of gait after ankle fracture, with the aim of outlining a common pattern of kinematic alterations. We were able to identify a few typical alterations related to trunk and lower limb kinematics. However, these findings are based on a relatively low number of studies, which are mainly case series involving small numbers of patients, with differences in terms of fracture type and treatment, rehabilitative protocols, time from injury, instrumental gait evaluation and clinical outcomes measured. Therefore, it is difficult to draw strong conclusions. Further studies are needed to evaluate the generalizability of our findings and to examine in greater depth the relation between gait alterations and possible rehabilitation interventions in the different categories of patients and phases of treatment.

### 4.4. Implications for Clinical Practice and Research

Our study identified several gait alterations after ankle fracture, which can be addressed through rehabilitation interventions. Based on our findings, we recommend the use of instrumental methods of gait assessment in clinical practice to objectively study the individual alterations after ankle fracture and guide selection of a personalized program of rehabilitation. In this context, it should be emphasized that gait kinematics differ between healthy people and patients in the short-term period after ankle fracture, so it is important to address gait alterations during the rehabilitation period to prevent the development of pathologic gait [32].

The identification of gait alterations may have significant consequences in the research field. Indeed, the objective definition of gait alterations after ankle fracture may be used to design future clinical trials as a reference point for the evaluation of existing or new rehabilitative interventions. This approach is instrumental to improving the quality of evidence produced in rehabilitation research [66].

## 5. Conclusions

The use of instrumental methods of gait assessment makes it possible to objectively determine gait alterations after ankle fracture, which can be of great help both in programming rehabilitation interventions as well as in designing future clinical trials.

## Figures and Tables

**Table 1:** Methodological quality using the MINORS scale for nonrandomized controlled trials.

	Items of the Scale								
Authors	1	2	3	4	5	6	7	8	Total
Elbaz et al. (2016) [32]	2	2	2	2	1	2	0	0	11
Hsu et al. (2019) [33]	2	2	2	2	1	2	2	2	15
Jansen et al. (2013) [34]	2	2	0	2	0	2	1	2	11
Quacinella et al. (2019) [35]	2	2	2	2	2	2	0	2	14
Segal et al. (2014) [36]	2	2	2	2	0	1	2	0	11
Suciu et al. (2016) [37]	2	2	2	2	2	2	2	2	16
Terrier et al. (2009) [38]	2	2	2	2	2	2	0	1	13
Terrier et al. (2013) [39]	2	1	2	2	2	2	0	2	12
Van Hoeve et al. (2019) [40]	2	2	2	2	2	2	2	2	16
Wang et al. (2010) [11]	2	1	2	2	0	2	0	0	9

Key for item 1–8: (1) clearly stated aim; (2) inclusion of consecutive patients; (3) prospective collection of data; (4) endpoints appropriate to the aim of the study; (5) unbiased assessment of the study endpoints; (6) follow-up time appropriate to the aim of the study; (7) loss to follow up less than 5% of patients; (8) prospective calculation of the sample size.

**Table 2:** Methodological quality using the PEDro scale for randomized controlled trials.

	Items of the Scale											
Authors	1	2	3	4	5	6	7	8	9	10	11	Total
Albin et al. (2019) [41]	Yes	Yes	Yes	Yes	Yes	No	Yes	Yes	Yes	Yes	Yes	9
Keene et al. (2016) [42]	Yes	Yes	Yes	Yes	No	No	No	No	Yes	Yes	Yes	6

Key for item 1–11: (1) specified eligibility criteria; (2) subjects randomly allocated to groups; (3) concealed allocation; (4) similar groups at baseline for the most important prognostic indicators; (5) blinding of all subjects; (6) blinding of all therapists who administered therapy; (7) blinding of all assessors who measured at least one key outcome; (8) measures of at least one key outcome obtained from more than 85% of the subjects initially allocated to groups; (9) all subjects for whom outcome measures were available received the treatment or control condition as allocated or, where this was not the case, data for at least one key outcome was analyzed by “intention to treat”; (10) results of between-group statistical comparisons reported for at least one key outcome; (11) indication of both point measures and measures of variability for at least one key outcome.

**Table 3:** Summary of the main information extracted from the selected articles.

	Subjects and Type of Fractures	Study Design	Orthosis	Device Used for Gait Analysis		Analysis	Functional Scales or Other Instrumental Evaluations	Main Results of Gait Analysis
Authors				Type	Location	Variables of Gait Assessed by the Device		
Albin et al. (2019) [41]	*n* = 72 patients with ORIF of an ankle and/or hindfoot fracture	Multisite double-blind randomized clinical trial. Manual therapy group (*n* = 40): impairment-based manual therapy. Control group (*n* = 32): sham manual therapy. Three treatment sessions over 7 to 10 days	None	Electronic pressure-sensitive walkway	N/A	Gait speed, Percent of time spent in single-limb support, Stance time	AOFAS Hindfoot Score, Lower Extremity Functional Scale (LEFS), Numeric Pain Rating Scale (NPRS), Beck Anxiety Inventory, Ankle Lunge Test for ankle dorsiflexion ROM, Foot Assessment Platform (FAP) for mid-foot mobility, gastrocnemius muscle stiffness, single-limb stance test (SLS) for balance, Star Excursion Balance Test (SEBT) for balance and reach	No difference in gait variables between the two groups.
Elbaz et al. (2016) [32]	*n* = 24 patients with unimalleolar, bimalleolar, or trimalleolar fracture and ≤6 weeks’ time from weight-bearing approval; *n*=24 healthy controls	Case-control	None	IMU on the thigh and calf of each leg	Lateral side of the calf and lateral side of the thigh	Knee ROM during the swing phase, Maximum knee flexion angle during stance, Thigh and calf ROM, Stride duration	None	Compared with controls, patients showed reduced knee ROM during swing phase, reduced maximum knee flexion angle during stance, lower gait cycle thigh and calf ROM, longer stride duration.
Hsu et al. (2019) [33]	*n* = 10 patients (median age 38 years) with unimalleolar, bimalleolar, trimalleolar ankle fracture treated with cast immobilization ± ORIF; *n* = 10 age- and sex-matched healthy controls	Case-control	None	Triaxial accelerometer	Lower back (L3–L4)	Walking speed, Step length, Cadence, Trunk movement symmetry and regularity, acceleration root mean square (RMS) in the AP, ML, V directions, and acceleration RMS ratio (RMSR) in the ML direction	Lower Extremity Functional Scale (LEFS), fall assessment during the 24 months after the fracture	Reduced walking speed, step length, and cadence in ankle fracture patients. Reduced trunk acceleration RMS in AP and VT directions in the ankle fracture group. Symmetry of trunk movement in the VT direction lower in the patient group. Positive rank correlation between acceleration RMSR in ML axis and future falls.
Jansen et al. (2013) [34]	*n* = 35 patients (average age 47.6 years) with unilateral pilon fracture surgically treated	Cross-sectional	None	Electronic pressure-sensitive walkway	N/A	Load, Pressure, Contact time during the roll-over process and force–time integral	Visual Analogue Scale (VAS) Foot and Ankle Questionnaire, American Orthopedic Foot and Ankle Society (AOFAS) questionnaire, Phillips score. Goniometry: ROM of the upper and lower ankle joint	Lesser load bearing for the total foot, medial foot, heel, first metatarsal and medial forefoot for the affected limb; increased load bearing in the lateral midfoot region.
Keene et al. (2016) [42]	*n* = 18 patients, aged 19–77 years who underwent internal fixation for transsyndesmotic/ infrasyndesmotic fracture	Randomized 3-treatment, 3-period (preferred walking speed, slow walking speed and fast walking speed) crossover trial to determine the immediate effects of different ankle supports to patients, 6 weeks after surgery	Elasticized compressive tubular bandage, or ankle stirrup brace, or removable below-knee walker boot	Electronic pressure-sensitive walkway	N/A	Walking speed, Step-length asymmetry, Single-limb support time asymmetry, Step width	Lower Extremity Functional Scale (LEFS), pain at rest and immediately after each ankle support using a visual analog scale (VAS), health state with the Health Utilities Index Mark 3, goniometry assessments of ankle active range of motion, handheld dynamometry of plantarflexion	Single-limb support time asymmetry reduced by 3% in the stirrup brace and by 5% in the walker boot compared with elasticized bandage. Step width 1.2 cm wider in the walker boot than in elasticized bandage.
Quacinella et al. (2019) [35]	*n* = 7 young subjects, pilon fractures	Between-subject comparison. Return-to-Run clinical pathway: 6-week-long program of three phases of gait retraining, strengthening, and agility utilizing the AFO (Ankle–Foot Orthosis) for assistance	Ankle–Foot Orthosis	Stereophotogrammetry, Floor-embedded force platforms	N/A	Gait speed, Cadence, Stride length, Single stance time	Pain (VAS) before and after AFO	Improvement of speed from 1.1 m/s to 1.3 m/s with AFO. Patient self-reported pain scores not changed
Segal et al. (2014) [36]	*n* = 41 patients (mean age 47.3 years) with uni-, bi- or trimalleolar fracture treated with ORIF and instructed to avoid weight bearing for 6 weeks; *n* = 72 healthy controls	Case-control	None	Electronic pressure-sensitive walkway	N/A	Gait speed, Involved and uninvolved step length, Involved and uninvolved single limb support	Foot and Ankle Outcome Score (FAOS), Short Form-36 Health Survey, American Orthopedic Foot and Ankle Score (AOFAS), 6 min walk test	Gait variables of all three fracture-severity groups significantly below the normal range. Significant differences between groups in all gait variables including gait speed, involved and uninvolved step length, involved and uninvolved single-limb support. Significant asymmetry in step length and single-limb support in all fracture groups.
Suciu et al. (2016) [37]	*n* = 30 patients with suprasyndesmotic bimalleolar fractures surgically treated with ORIF; *n* = 21 healthy controls	Two assessments for the study group: the first one—once the weight-bearing was allowed (6–8 weeks after surgery—T1) and the second one—twelve weeks after exercise-based rehabilitation program (T2)	None	Treadmill with an integrated electronic pressure-sensitive platform	N/A	Speed, Cadence, Step length, Stride length, Percent of time spent in Step time, Stance time, Swing time, Load response time, Pre-swing time, Single support time	Olerud–Molander Ankle Score (OMAS)	In T1, significant differences in all temporal and spatial gait variables between the patient group and controls. In T2, no significant changes between patients and controls in step time in the affected ankle and nonaffected ankle, swing time and stance time in the affected ankle, or stride time and cadence. Step time, step length, stance, swing and single support significantly shorter in the affected than nonaffected leg in T1 and T2
Terrier et al. (2009) [38]	*n* = 16 patients (mean age 44.7 years) with ankle and/or foot fractures (mean time postinjury 18.4 months); *n* = 16 healthy male subjects	Case-control	Orthopedic shoes: low shoe or ankle boot	Triaxial accelerometer	Lower back (L3–L4)	Stride Symmetry, Stride Regularity	Visual Analog Scale (VAS), AOFAS (American Orthopedic Foot and Ankle Society) Hindfoot or Midfoot Questionnaires	Greater stride symmetry and stride regularity with than without prescription footwear. Without prescription footwear, stride symmetry of patients lower than that of healthy subjects. Stride regularity of patients not statistically different from that of healthy subjects.
Terrier et al. (2013) [39]	*n* = 25 patients (mean age 48 years), about 8 months after injury	Within subjects	Orthopedic shoes: low shoe or ankle boot	Triaxial accelerometer	Lower back (L3–L4)	Stride regularity, Stride symmetry	American Orthopedic Foot and Ankle Society (AOFAS), Hindfoot or Midfoot Questionnaires, Visual Analogue Scale (VAS)	Higher local dynamic stability of walking with than without orthopedic shoes. Larger stability in the mediolateral direction. Cadence unchanged.
Van Hoeve et al. (2019) [40]	*n* = 33 patients (age range 25–78 years) surgically treated for unstable ankle fracture immobilized with cast for 6 weeks and evaluated at 18-month follow up; *n* = 11 healthy subjects	Case–control	None	Stereophotogrammetry, Force platform embedded in a 10 m walkway	Markers placed according to the Oxford Foot Model (OFM)	ROM between hindfoot and tibia in the frontal, sagittal and transverse planes	Foot and Ankle Disability Index (FADI), Visual Analogue Scale (VAS), American Orthopedic Foot and Ankle Society (AOFAS) hindfoot-ankle score, Short-Form 36 score (SF-36)	Significant difference in walking speed between the two groups when patients walked at preferred normal speed but not when healthy subjects walked slowly and the ankle fracture patients walked at normal speed. When adjusted for speed, lower ROM between the hindfoot and tibia in the sagittal plane (flexion/extension) during both the loading and push-off phases lower in the ankle group than among the healthy subjects. No significant differences between the two groups in the ROM in the frontal plane (abduction/adduction) and transverse plane (inversion/eversion), nor during the loading phase or during the push-off phase. Significantly lower ROM between hindfoot and tibia in the sagittal plane (flexion/extension) during the push-off phase in the patients with a trimalleolar ankle fracture compared to patients with a unimalleolar fracture.
Wang et al. (2010) [11]	*n* = 18 patients (aged 17–64 years) with lateral malleolar or trimalleolar fractures 1 year after ORIF; unspecified number of healthy controls	Case–control	None	Markers, Camera motion system	Markers placed according to the modified version of the Oxford Foot Model: tibia, hindfoot, forefoot and hallux (bilateral)	Hindfoot/Tibia, Forefoot/Hindfoot, Forefoot/Tibia, Hallux/Forefoot kinematics during gait	Olerud–Molander ankle score (OMAS)	Reduced plantarflexion and range of motion in the injured ankle joint during swing phase. Decreased sagittal and transverse ranges of motion in both stance and swing phase and reduced plantarflexion of the forefoot in swing phase. Reduced dorsiflexion and sagittal range of motion of the hallux segment in swing phase. Reduced single support time on the injured side. With respect to healthy subjects, shorter stride length and delayed foot-off time in the noninjured side.

Abbreviations: ROM, range of motion; IMU, inertial measurement unit; AP, anteroposterior; ML, mediolateral; V, vertical; ORIF, open reduction and internal fixation; N/A, not applicable.

**Table 4:** List of domains and relative clinical outcome measures evaluated in the selected articles.

	Instrument	Measured Variable	Studies
	Evaluator-Based Scoring Systems		
**Range of Motion**	Ankle lunge test (ALT)	Weight-bearing ankle dorsiflexion ROM	Albin et al. (2019) [41]
	Foot Assessment Platform (FAP)	Midfoot mobility (medial–lateral and vertical)	Albin et al. (2019) [41]
	The score of Phillips	Ankle and subtalar joint ROM, ankle stability, inflammation (synovitis), pressure pain, radiological signs of osteoarthritis	Jansen et al. (2013) [34]
	Goniometer	Active dorsiflexion, plantar flexion	Keene et al. (2016) [42], Segal et al. (2014) [36], Jansen et al. (2013) [34]
	American Orthopedic Foot and Ankle Society (AOFAS) Ankle–Hindfoot Score	Pain, function, alignment	Albin et al. (2019) [41], Jansen et al. (2013) [34], Segal et al. (2014) [36], Terrier et al. (2009) [38], Terrier et al. (2013) [39], Van Hoeve et al. (2019) [40]
**Balance**	Single-limb stance test (SLS)	Static balance, postural control	Albin et al. (2019) [41]
	Star Excursion Balance Test (SEBT)	Dynamic balance	Albin et al. (2019) [41]
**Gait**	6 min walking test	Endurance	Segal et al. (2014) [36]
	**Patient-reported measures**		
**Impairment, Disability, Participation**	Visual analogue scale (VAS)	Pain at rest or during movement	Jansen et al. (2013) [34], Keene et al. (2016) [42], Terrier et al. (2009) [38], Terrier et al. (2013) [39], Van Hoeve et al. (2019) [40]
	Visual analogue scale for foot and ankle (VAS FA)	Pain, function, other complaints	Jansen et al. (2013) [34]
	American Orthopedic Foot and Ankle Society (AOFAS) Ankle–Hindfoot Score	Pain, function, alignment	Albin et al. (2019) [41], Jansen et al. (2013) [34], Segal et al. (2014) [36], Terrier et al. (2009) [38], Terrier et al. (2013) [39], Van Hoeve et al. (2019) [40]
	Numeric Pain Rating Scale (NPRS)	Pain intensity	Albin et al. (2019) [41], Quacinella et al. (2019) [35]
	Foot and Ankle Outcome Score (FAOS)	Symptoms, stiffness, pain, function (daily living), sport and recreational activities, quality of life	Segal et al. (2014) [36]
	Olerud–Molander Ankle Score (OMAS)	Pain, stiffness, swelling, stair climbing, running, jumping, squatting, supports and activities of daily living	Suciu et al. (2016) [37], Wang et al. (2010) [11]
	The score of Phillips	Pain, stability, walking, activity level and sport, walking distance, swelling, weather influence	Jansen et al. (2013) [34]
	The Foot & Ankle Disability Index (FADI) Score	Functional limitations related to foot and ankle conditions	Van Hoeve et al. (2019) [40]
**Activities of Daily Living**	Lower Extremity Functional Scale (LEFS)	Ability to perform everyday tasks	Albin et al. (2019) [41], Hsu et al. (2019) [33], Keene et al. (2016) [42]
**Quality of life**	36-Item Short Form Health Survey questionnaire (SF-36)	Vitality, physical functioning, bodily pain, general health perceptions, physical role functioning, emotional role functioning, social role functioning, mental health	Segal et al. (2014) [36], Van Hoeve et al. (2019) [40]
**Psychological symptoms**	Beck Anxiety Inventory (BAI)	Severity of anxiety	Albin et al. (2019) [41]

## Data Availability

Data supporting the reported results can be found at PubMed (National Library of Medicine), Embase (Ovid) and Pedro databases.
